# How Does Green Training Boost Employee Green Creativity? A Sequential Mediation Process Model

**DOI:** 10.3389/fpsyg.2021.759548

**Published:** 2021-12-08

**Authors:** Jianfei Wu, Dan Chen, Zejuan Bian, Tiantian Shen, Weinan Zhang, Wenjing Cai

**Affiliations:** ^1^Ance Think Tank, Hefei, China; ^2^International Institute of Finance, School of Management, University of Science and Technology of China, Hefei, China; ^3^Intellectual Property Research Institute, University of Science and Technology of China, Hefei, China; ^4^School of Public Affairs, University of Science and Technology of China, Hefei, China; ^5^Department of Management and Organization, Vrije Universiteit Amsterdam, Amsterdam, Netherlands

**Keywords:** green training, green values, green intrinsic motivation, green creativity, sequential mediation

## Abstract

Despite accumulated evidence from previous studies that green creativity is highly emphasized in various industries, limited research has been conducted in the context of public sectors. Drawing on the dynamic componential model of creativity and innovation in organizations, this paper aims to propose and sequentially test the relationship between green training and employees’ green creativity through green values and green intrinsic motivation. Based on the data collected in Chinese public sectors (*N* = 464) at two different time points, the results indicate that green training is positively related to green creativity. Moreover, this relationship is sequentially mediated by green values and green intrinsic motivation. The results in our study advance the emergent literature on green human resource management in the public sector for the practical applications of training and creativity in terms of green management.

## Introduction

With the increasing acknowledgments of boosting individuals’ creativity in the workplace, practitioners and scholars have recently devoted attention to green creativity issues (Chen and Chang, 2013; Song and Yu, 2018) because global activities concerned with environmental issues and organizations benefit from a wide range of creative options in a green manner (Mittal and Dhar, 2016). However, the topic of green creativity in the public sector has been largely overlooked, perhaps due to the overwhelming focus on profit organizations (e.g., services and manufacturing sectors) in previous research. Given global challenges (e.g., the current scarcity of natural resources), some scholars have called for green outputs in public sectors to be investigated because green public sector strategies have been adopted internationally by offering green services (Boenigk and Möhlmann, 2016).

However, limited research has been conducted to examine green creativity among employees in the public sector. The current paper aims to fill this research gap by introducing green training as an important predictor. Specifically, as the most widely followed green practice (Jabbour, 2013), green training in the workplace can build a sound working environment characterized by highlighting green issues and philosophy (Talan and Sharma, 2019). Existing studies have indicated the extent of green training by empirically showing that employees who receive training on green issues develop their environmental knowledge and awareness of initiating green-oriented activities (Teixeira et al., 2012; Obaid and Alias, 2015). Following this line of literature, we further expect that green training contributes to employees’ green creativity.

According to the theoretical arguments of the dynamic componential model of creativity and innovation in organizations (Amabile and Pratt, 2016), when primarily intrinsically motivated, individuals are more likely to produce creative outcomes in the workplace. The theoretical reasoning lies in the fact that the social environment (e.g., working context) influences employee creativity largely through its impact on individuals’ intrinsic motivation, which can be defined as an individual’s internal motivation to react to tasks positively (Amabile, 1996). Following this line of reasoning, we expected that green training boosts employees’ creativity by developing their intrinsic green motivation.

**Figure 1:**
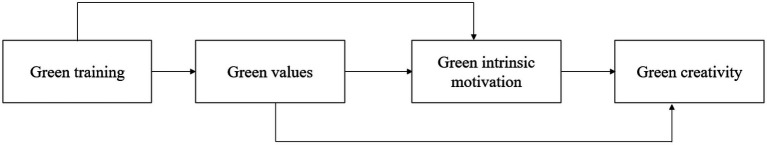
The hypothesized model.

The dynamic componential model of creativity and innovation in organizations further suggests the process perspective in the creativity literature—that is, the dynamism of the process of creativity. Specifically, creative employees’ well-developed creativity-relevant processes (e.g., mindset of creative engagement) are linked with strong intrinsic motivation toward the higher level of creative ideas generated (Amabile and Pratt, 2016). As scholars in a recent review paper (Anderson et al., 2014) claimed to reinvigorate process research in the creativity literature, we propose that green values act as a mediator to link green training and employees’ green intrinsic motivation. Specifically, green training as a contextual stimulus has been evidenced to trigger individuals’ corresponding attitudes and behavior, such as developing green values (e.g., Deng et al., 2013). Therefore, their green values are likely to boost their motivations to engage in environmental endeavors intrinsically (e.g., Li et al., 2020), which in turn results in green output in a creative manner. Taken together, we propose and test that green training contributes to employees’ green creativity by sequentially increasing their green values and intrinsic green motivation. Figure 1 shows our hypothesized model.

By employing the dynamic componential model of creativity and innovation in organizations, the current study aims to link green training to green creativity by exploring the chain mechanism in the public sector. First, we identified green training in organizations toward increasing the level of green creative outputs. In doing so, we extend the current understanding of cultivating employees’ green creativity by exploring a wide range of predictors (i.e., green training). Second, by applying the dynamic componential model of creativity and innovation in organizations, the current study unfolds the sequential process (i.e., green value and green intrinsic motivation) of the association between green training and green creativity. Thus, we address the call for a better understanding of contextual factors (i.e., green training) from the process perspective. Finally, we enrich the knowledge of green creativity in the special context of public sectors by highlighting the importance of green creativity theoretically and the practices of stimulating employee creativity in a green manner practically.

## Theory and Hypotheses

### Green Training and Green Creativity

Considering the key role of the “human dimension” in successfully enacting environmental and green activities in modern organizations (Renwick et al., 2013; Jabbour et al., 2019), an emerging stream of research has adopted a “behavioral perspective” on “green” HRM practices by stressing green training to employees throughout organizations (Jabbour, 2013; Teixeira et al., 2016). Conceptually, a process of on-the-job training and continued education is designed to achieve corporate environmental management targets and purposes (Daily and Huang, 2001).

According to previous research, green training is prioritized as the most effective human resource management practice promoting superior environmental performance (Renwick et al., 2013) since training programs can motivate participants’ formal awareness; that is, employees are aware of informal practices, such as energy conservation to voluntarily reciprocate green behavior (Stefanelli et al., 2021). Consistently, some scholars have evidenced that green training can contribute to extrarole behaviors (Pinzone et al., 2019) because it can build a supportive working context through appropriate interventions toward successful implementation of green goals (Dumont et al., 2017).

As green creativity, referring to developing original, novel, and useful ideas about green products, green services, green processes, or green practices (Chen and Chang, 2013), is recognized as a typical extrarole behavior in organizations, we follow previous studies and propose that green training can stimulate employees’ green creativity. Specifically, when organizations provide green training programs, they intuitively signal that creative green behaviors are expected and valued throughout the organization (Obaid and Alias, 2015). As mentioned above, green training formulates a desirable working situation in favor of green activities. In this situation, the diffusion of environmental awareness and competencies from green training helps employees become better able to take suitable actions to mitigate environmental impacts in the workplace. As a result, they adopt new “green” behaviors in a creative manner to meet the green requirements of organizations. Therefore, we propose the following hypothesis:

*Hypothesis 1:* Green training is positively related to employees’ green creativity.

### The Mediator of Green Values

We propose the positive effect of green training on employees’ green values due to the value-based nature of green training. Studies have suggested that the principal purpose is to deliver the value of green in organizations to employees (Amrutha and Geetha, 2021). That is, employees who are trained in proper strategies of behaving in a green manner inevitably develop green-oriented values in the workplace. In addition, receiving green training can increase employees’ awareness and cognition of organizational green values (Pinzone et al., 2019), which in turn reinforces employees’ self-values regarding being green (Wang et al., 2018).

We expect a positive association between green values and green creativity according to the current literature, which has acknowledged that personal values can influence individual attitudes and behavior (Al-Ghazali and Afsar, 2021; Kalyar et al., 2021). Empirical studies (Andersson et al., 2005; Schultz et al., 2005) have reported a significant and positive impact of personal environmental values on individual environmentally friendly behavior. Relating to our research context, we integrate our theoretical argumentations with supplies-values fit theory (Edwards, 1996). That is, when an organization provides an environment that is conducive to the development of employee values (i.e., green training), employees’ green values are consistent with the organization. In this situation, employees will have creative ideas to solve the green problem (Cohen and Liu, 2011; Al-Ghazali and Afsar, 2021).

Based on the reasoning above and Hypothesis 1, we expect that when organizations provide training programs on green issues to employees, these employees sense the necessities and valuableness of green activities in the workplace. Consequently, they are more likely to engage in producing green creative outcomes. Taken together, we propose the following hypothesis:

*Hypothesis 2:* Green values mediate the relationship between green training and employees’ green creativity.

### The Mediator of Green Intrinsic Motivation

Adapting the dynamic componential model of creativity and innovation in organizations (Amabile and Pratt, 2016), we argue, first, that green training can trigger employees’ green intrinsic motivation. Green intrinsic motivation was developed from the concept of intrinsic motivation, which represents a state in which people are internally motivated by enthusiasm for performing the tasks (Deci and Ryan, 1985). Scholars have recently highlighted the concept of green intrinsic motivation by suggesting that desirable contexts are significant predictors of individuals’ green intrinsic motivation (Li et al., 2020), as they help employees sense interests and feel excited about the content of their work (Oldham and Cummings, 1996; Shalley et al., 2004). Regarding green tasks, since green training signals an environment of supporting green attitudes and behaviors, employees perceive green training as a form of organizational investment in their personal growth and development of doing green tasks. As a result, their intrinsic motivation to take green responsibilities are increased.

We further expect a positive effect of green intrinsic motivation on employees’ green creativity. Theoretically, employees are strongly intrinsically motivated to find work assignments interesting because they are internally stimulated to put effort into completing tasks. In terms of working in the green context, employees who have received green-related training will be intrinsically motivated for green tasks through a desire to display creative behaviors. The main reason is that employees with a high level of green intrinsic motivation are naturally interested in environmental issues, which in turn enables them to better enjoy working on green assignments and projects (Amabile, 1997; Deci and Ryan, 2016). In this situation, their inspirations are effectively stimulated to generate creative ideas to address green problems.

According to the reasoning above and the proposed Hypothesis 1, we expect intrinsic green motivation to mediate the relationship between green training and green creativity. Specifically, the more training an organization offers, the greater green intrinsic motivation can be developed, and consequently, employees’ green creative products can be generated. Therefore, we propose the following hypothesis:

*Hypothesis 3:* Green intrinsic motivation mediates the relationship between green training and employees’ green creativity.

### The Sequential Mediating Effects of Green Values and Green Intrinsic Motivation

As the dynamic componential model of creativity and innovation in organizations suggests that creativity is a process that contains some personal attributes (e.g., cognitive and motivational factors; Amabile and Pratt, 2016), we propose that green values and green intrinsic motivation sequentially mediate the relationship between green training and employee green creativity. Specifically, considering the research evidence that green training indirectly affects employees’ green creativity, employees who participate in green training courses aim to develop a sense that engaging in green activities is highly valuable in organizations. In such situations, they are more likely to generate internal motivations to implement green philosophy in their tasks, which in turn leads to more green creative outcomes. Integrating the dynamic componential model of creativity and innovation in organizations with the discussion and reasoning above, we propose the last hypothesis:

*Hypothesis 4:* Green values and green intrinsic motivation sequentially mediate the relationship between green training and employees’ green creativity.

## Materials and Methods

### Sample and Procedure

We employed the research design of survey questionnaires in the present study. Since one of the research aims is to examine green creativity in the public sector, we invited employees and their direct leaders working in the public sector in China to complete the questionnaires. Before submitting our questionnaires, the authors randomly contacted individuals in twenty public sectors and informed them about the contents of the present study. After receiving confirmation from individuals in nine public sectors that indicated that they were encouraging green creativity in their own organizations, we randomly selected 800 employees in these public sectors and then submitted the first set of questionnaires to them at Time 1. These employees were informed that their responses would be kept confidential. Meanwhile, we asked them to report the name of their direct supervisors, who would rate their (i.e., employees) green creativity at Time 2. They rated green training, green values and green intrinsic motivation and provided some demographic information. We received 668 responses after deleting responses with incomplete information. After 1 month, at Time 2, we submitted the other set of questionnaires to these employees’ direct leaders according to these employees’ reports at Time 1. We asked these leaders to rate their followers’ green creativity. After deleting responses with incomplete information, we finally received 464 valid responses.

Among these 464 employees in the public sector, 53.2% were female (SD = 0.50), and their average age was 30.72 (SD = 7.11). The most frequently reported education level was associate degree (50.2% employees, SD = 0.71). Their average work tenure in the current organization was 2.82 (SD = 2.10).

### Measures

#### Green Training

We used the 6-item scale from Pham et al. (2019) to measure green training at Time 1 (e.g., “Employees have many opportunities to use environmental training”; Cronbach *α* = 0.859). The KMO value was 0.729, with the Bartlett test of sphericity achieving statistical significance (*p* < 0.001).

#### Green Values

The five-item scale from Chou (2014) was used to assess green values at Time 1 (e.g., “I feel a personal obligation to do whatever I can to prevent environmental degradation”; Cronbach *α* = 0.944). The KMO value was 0.873, with the Bartlett test of sphericity achieving statistical significance (*p* < 0.001).

#### Green Intrinsic Motivation

We asked green intrinsic motivation with six items from Amabile et al. (1994) at Time 1 (e.g., “I enjoy coming up with new green ideas”; Cronbach *α* = 0.845). The KMO value was 0.831, with the Bartlett test of sphericity achieving statistical significance (*p* < 0.001).

#### Green Creativity

At Time 2, green creativity was measured with six items from Chen and Chang (2013; e.g., “The members of the organization would rethink new green ideas”; Cronbach *α* = 0.943). The KMO value was 0.731, with the Bartlett test of sphericity achieving statistical significance (*p* < 0.001).

#### Control Variables

We controlled the following variables: gender (1 = male; 2 = female), age (in years), educational level (1 = High school/technical school and below; 2 = Bachelor’s degree; and 3 = Master’s degree and above), and working tenure (1 = less than 1 year; 2 = from 1 to 5 years; 3 = from 6 to 10 years; and 4 = more than 10 years).

### Analytical Strategy

Before testing hypotheses, we first used SPSS software version 25 to analyze the data. Specifically, we calculated the descriptive statistics to characterize all the variables in the current study—computing Pearson’s product–moment correlation to test the directions and correlations among all the variables. To test our hypothesis that green values and green intrinsic motivation act as serial mediators of the relationship between green training and green creativity, we used the SPSS PROCESS macro, Model 6, to test the stability and significance of the mediation effects. Particularly, we calculated 95% confidence intervals of the indirect effects derived from bias-corrected bootstrap estimates with 5,000 iterations, which are significant at *p* = 0.05 if the 95% confidence interval does not include zero. We employed PROCESS to test our hypotheses because it is widely used in the social, business, and health sciences to estimate direct and indirect effects in single and multiple mediation models (Hayes and Scharkow, 2013; Solove et al., 2015; Baroudi et al., 2018). PROCESS generates all of the statistical calculations and implements bootstrapping in a way that facilitates inference about moderated and mediated effects (Hayes and Scharkow, 2013; Hayes et al., 2017). In the current study, specifically, we used Model 6 to perform a sequential mediation analysis that explicitly tested how the in-dependent variable (i.e., green training) can influence the dependent variable (i.e., green creativity) by influencing two distinguished mediators in a sequential way (i.e., influencing green values and then green intrinsic motivation).

## Results

### Preliminary Analyses

Before testing hypotheses, we conducted confirmatory factor analysis (CFA) to establish the discriminant validity of all the factors. We employed AMOS 25.0 and showed the results in Table 1. Specifically, the results of all the alternative models are as follows: the three-factor model (*χ*^2^ = 1572.768, *df* = 222, *χ*^2^/*df* = 7.085, RMSEA = 0.115, GFI = 0.719, TLI = 0.824, CFI = 0.846); the two-factor model (*χ*^2^ = 3206.299, *df* = 225, *χ*^2^/*df* = 14.250, RMSEA = 169, GFI = 0.537, TLI = 0.617, CFI = 0.660); and the one-factor model (*χ*^2^ = 5123.279, *df* = 229, *χ*^2^/*df* = 22.372, RMSEA = 0.215, GFI = 0.420, TLI = 0.383, CFI = 0.442). Comparatively, the results of our hypothesized four-factor model indices are *χ*^2^ = 575.489, *df* = 218, *χ*^2^/*df* = 2.640, RMSEA = 0.060, GFI = 0.904, TLI = 0.953, CFI = 0.959. Therefore, our hypothesized model showed a better fit than all the alternative models (Hu and Bentler, 1999).

**Table 1:** Comparison of measurement models.

Model	*χ*^2^ (*df*)	RMSEA	GFI	TLI	CFI
Four factors (baseline model): Green training, green values, green intrinsic motivation and green creativity	575.489 (218)	0.060	0.904	0.953	0.959
Three factors: Green values and green intrinsic motivation combined	1572.768 (222)	0.115	0.719	0.824	0.846
Two factors: Green training, green values and green intrinsic motivation combined	3206.299 (225)	0.169	0.537	0.617	0.660
One factor: All variables combined	5123.279 (229)	0.215	0.420	0.383	0.442

Table 2 shows the means, standard deviation, and correlations of all the measures. The results show that the relationship between green training and green creativity was significant (*β* = 0.254, *p* < 0.001), providing initial support for H1. As discussed, green training correlates to green values (*β* = 0.280, *p* < 0.001) and green intrinsic motivation (*β* = 0.468, *p* < 0.001). Moreover, both green values (*β* = 0.540, *p* < 0.001) and green intrinsic motivation (*β* = 0.388, *p* < 0.001) correlate to green creativity.

**Table 2:** Descriptive statistics and correlations between variables.

S. No.	Variables	Mean	SD	1	2	3	4
1.	Green training	4.927	0.918	(0.859)			
2.	Green values	5.250	1.179	0.280^***^	(0.944)		
3.	Green intrinsic motivation	5.062	0.835	0.468^***^	0.369^***^	(0.845)	
4.	Green creativity	5.037	0.977	0.243^***^	0.540^***^	0.388^***^	(0.943)

*N = 464. The diagonal is the Cronbach’s alpha*.

*** *p < 0.001*.

### Hypotheses Testing

**Figure 2:**
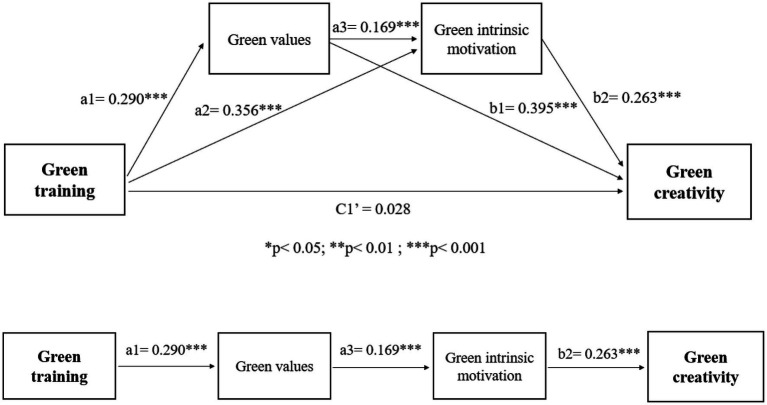
The results of the sequential model with path coefficients.

To test the hypothesis that green values and green intrinsic motivation sequentially mediate the impact of green training on green creativity, we performed a sequential mediation analysis (Model 6, as described in PROCESS) with bootstrap methods (Hayes, 2013). Figure 2 describes all the paths for the full process model. Table 3 shows the coefficients. The results indicate that the total effect (C1) of green training on leader-rated green creativity was found to be significant (*β* = 0.249, *t* = 5.017, *p* < 0.001), supporting H1. Moreover, the results in Table 3 show that the total direct effect (C1′) was insignificant (*β* = 0.028, *t* = 0.047, *p* > 0.05). The total indirect effect (i.e., the sum of the specific indirect effects) was significant, with a total indirect effect (*β* = 0.221, SE = 0.036) and a 95% confidence interval between 0.155 and 0.294.

**Table 3:** Results of sequential mediation analyses (PROCESS Model 6 in SPSS).

Model 6						
Y = Green creativity						
X = Green training						
M1 = Green values						
M2 = Green intrinsic motivation						
Covariates:						
Age Gender Tenure Education						
Sample size: 484						
Outcome:	Green values					
Model 1:	Summary					
	*R*	R-sq	*F*	Df1	Df2	*p*
	0.371	0.138	14.655	5	458	0.000
	Coefficient		SE	*t*	*p*	
Constant	3.424		0.425	8.063	0.000	
Green training	0.290		0.058	5.036	0.000	
Age	0.026		0.008	3.231	0.001	
Gender	0.153		0.103	1.485	0.138	
Tenure	−0.004		0.028	−0.135	0.892	
Education	−0.308		0.075	−4.100	0.000	
Outcome:	Green intrinsic motivation					
Model 2:	Summary					
	*R*	R-sq	*F*	Df1	Df2	*p*
	0.547	0.299	32.536	6	457	0.000
	Coefficient		SE	*t*	*p*	
Constant	1.840		0.290	6.343	0.000	
Green training	0.356		0.038	9.435	0.000	
Green values	0.169		0.030	5.641	0.000	
Age	0.018		0.005	3.367	0.001	
Gender	0.016		0.066	0.245	0.807	
Tenure	−0.012		0.018	−0.689	0.491	
Education	0.024		0.049	0.482	0.630	
Outcome:	Green creativity					
Model 3:	Summary					
	*R*	R-sq	*F*	Df1	Df2	*p*
	0.585	0.342	33.832	7	456	0.000
	Coefficient		SE	*t*	*p*	
Constant	1.689		0.344	4.917	0.000	
Green training	0.028		0.047	0.601	0.548	
Green values	0.395		0.035	11.255	0.000	
Green intrinsic motivation	0.263		0.053	4.956	0.000	
Age	−0.013		0.006	−2.119	0.035	
Gender	−0.021		0.075	−0.277	0.782	
Tenure	0.023		0.020	1.168	0.244	
Education	0.080		0.056	1.442	0.150	
Outcome:	Green creativity					
Model 4:	Summary					
	*R*	R-sq	*F*	Df1	Df2	*p*
	0.254	0.064	6.306	5	458	0.000
	Coefficient		SE	*t*	*p*	
Constant	3.677		0.367	10.028	0.000	
Green training	0.249		0.050	5.017	0.000	
Age	0.003		0.007	0.483	0.629	
Gender	0.051		0.090	0.570	0.569	
Tenure	0.019		0.024	0.779	0.436	
Education	−0.050		0.065	−0.755	0.451	
Total, direct, indirect effects						
Total effects of green training on green creativity						
	Effect		SE	*t*	*p*	
	0.249		0.050	5.017	0.000	
Direct effects of green training on green creativity						
	Effect		SE	*t*	*p*	
	0.028		0.047	0.601	0.548	
Indirect effects of green training on green creativity						
	Effect		Boot SE	BootLLCI	BootULCI	
Total:	0.221		0.036	0.155	0.294	
Ind 1:	0.114		0.026	0.067	0.169	
Ind 2:	0.094		0.023	0.052	0.143	
Ind 3:	0.013		0.005	0.005	0.026	
Indirect effect key						
Ind 1:	Green training → green values → green creativity					
Ind 2:	Green training → green intrinsic motivation → green creativity					
Ind 3:	Green training → green values → green intrinsic motivation → green creativity					
Analysis notes						
Bootstrap samples for bias-corrected bootstrap confidence intervals: 5,000						
Level of confidence for all confidence intervals in output: 95%						
BootLLCI = lower limit confidence interval; BootULCI = upper limit confidence interval						

Moreover, the specific indirect effect resulting from green values only was significant (a1b1 = 0.114; 95% CI = 0.067 and 0.169), and the specific indirect effect resulting from green intrinsic motivation was significant (a2b2 = 0.094; 95% CI = 0.052 and 0.143). The results indicated that both H2 and H3 are supported.

To test the sequential multiple mediation effect (i.e., H4), the results showed that the specific indirect effect of green training on leader-rated green creativity through both green values and green intrinsic motivation (a1a3b2) was significant, with a point estimate of 0.013 and a 95% confidence interval between 0.005 and 0.026, providing full support for H4. Therefore, green training might lead to positive green values, which might be a unique predictor to increase the level of green intrinsic motivation, and green intrinsic motivation increases leader-rated green creativity, as our hypothesis is supported in the current study. Taken together, the results prove that green values and green intrinsic motivation sequentially mediate the linkage between green training and leader-rated green creativity. In this vein, it is suggested that after receiving trainings on green and environmental issues, employees develop their green values and green intrinsic motivation sequentially; as a result, their leaders evaluate their green creativity in a positive way with higher scores.

## Discussion

### Theoretical Implications

First, this was among the first studies to investigate the possibilities of whether and how green training can promote green creativity in employees. We found that when organizations provide training programs related to green topics, employees generate high levels of green creativity in the workplace (Jabbour, 2013). Specifically, organizations offering green training can create a sustainable working environment where employees feel encouraged to engage in producing green solutions in a creative manner (Renwick et al., 2013; Stefanelli et al., 2021). As such, our results reinforce the need to reconsider the role of green human resource management in modern organizations by highlighting the applications of training courses. Furthermore, we enriched the current understanding of the predictors of cultivating employees’ green creativity in organizations, that is, providing green training. In this vein, we contribute to the practical applications of training and creativity in terms of green management (Wang et al., 2013).

Second, we fed into a growing stream of studies that apply the dynamic componential model of creativity and innovation in organizations in the creativity literature (Amabile and Pratt, 2016). Although creativity scholars following the dynamic componential model of creativity and innovation in organizations have provided empirical evidence that contextual factors facilitate employee creativity by promoting his or her intrinsic motivation, limited studies have employed these findings in the green literature (Ogbeibu et al., 2021). We uniquely moved beyond these findings to empirically test the green intrinsic motivation that links a specific contextual factor (i.e., green training) and green creativity (Farooq et al., 2021). That is, we found that green training boosts employees’ green intrinsic motivation, which in turn leads to green creativity.

In addition, we empirically tested the dynamic componential model of creativity and innovation in organizations by identifying the other mediator of green values. That is, green training can contribute to green creativity *via* green values. This is consistent with the theoretical argument that some personal cognitive characteristics may transfer the benefits of desirable contexts in creativity (Shalley et al., 2004). Meanwhile, by unfolding the sequential process (i.e., green value and green intrinsic motivation) of the association between green training and green creativity, we addressed scholars’ call for investigations of the process perspective in the creativity literature. That is, situational factors (e.g., green training) can exert positive effects on an individual’s cognitive and motivational attributes toward the development of creativity. In addition, our assessment of green creativity is based on employees’ direct supervisors, which increases the objectiveness of measures. In this vein, we extend the previous creativity literature (George and Zhou, 2001; Reiter-Palmon et al., 2012) to green-related creativity research by using supervisor ratings of green creativity, which are the most common method in field studies and give more objective results than the self-rating method.

Finally, our study provides an in-depth understanding of managing employees’ green creativity in the public sector. Existing research stresses that green strategy is highly recognized in modern organizations, and investigations in the context of public sectors are extremely rare (Green and Roberts, 2012). This emphasis leads to a call for a burgeoning interest in understanding how to cultivate green creativity in the public sector. The current study contributed to the practices of stimulating employee creativity in a green manner, especially in the public sector. Since green strategy is highly recognized in modern organizations, our findings validate the importance of managing green behaviors throughout the public sector and can be generalized to other sectors that require environmentally oriented attitudes and behaviors in a creative way (e.g., Cai et al., 2020; Arici and Uysal, 2021). Moreover, relating to our highlighted green trainings in public sectors, it theoretically extends the current literature by suggesting that contemporary business organizations update managerial philosophy to invest more effort into training employees who are green creative (e.g., Pinzone et al., 2019). In this vein, HR departments are encouraged to provide both formal and informal training programs to enhance staff’s green and creative mindsets (e.g., Liu et al., 2020).

### Practical Implications

The findings of the current study have some practical implications. First, given the benefits of green training, organizations, especially in the public sector, should adopt a series of green training programs to develop employees’ creative green outputs. In doing so, employees can be oriented to the organizational stance on sustainability issues and what the expectations are for them to further green efforts. For example, HR departments should first try to receive enough information about employees’ needs to take training courses related to green and environmental issues in the workplace. Afterward, relevant training programs can be designed and provided accordingly (Shah, 2019). In this way, employees can be effectively equipped with the necessary skills and expertise for the successful implementation of green management goals in a creative way. Second, organizations should properly appraise employees’ green creative endeavors and link this behavior to promotional opportunities, pay, and compensation to encourage and motivate employees to participate in green activities and to contribute to green management objectives. For example, monthly and/or quarterly evaluations should be made, and then, related feedback should be provided to employees, which enables employees to receive a comprehensive picture of their green-oriented performance (Shou et al., 2020) and then make more efforts to behave creatively in an environmental manner. Third, managers can take responsibility to encourage employees to create a value of being green and environmental. Meanwhile, by assigning tasks requiring green creativity to employees, managers can stimulate their followers’ intrinsic motivations to develop creative ideas and solutions through a green method. For example, when providing feedback, supervisors can signal to employees that green creativity is highly valued and encouraged.

### Limitations

Some limitations of the present study should be noted. The first limitation regards the time-lagged research design used to collect data at two different time points. Despite the independence of variables measured at specific and different time points, their substantial correlations can be effectively shown when analyzed longitudinally. Thus, forthcoming longitudinal studies are recommended to replicate and extend our results. Second, we collected data from public sectors in China; thus, our research findings should be generated by conducting research in other countries. Finally, in addition to exploring the chain mechanism through which green training contributes to employees’ green creativity, research in the future should identify the boundary conditions to further understand the relationship between green training and green creativity.

## Conclusion

Drawing on the dynamic componential model of creativity and innovation in organizations, the current study aims to extend the understanding of green training and employees’ green creativity. By identifying the chain mechanisms—that is, green values and green intrinsic motivation—sequentially mediate the green training-green creativity association. Thus, we unfold the black box of why and how green training provided by organizations can contribute to increasing employees’ green output in a creative way. Moreover, as we conducted the research in the context of public sectors, our results add value to the pertinent literature on green human resource management in the public sectors, offering that environmental management practices improve managerial principles that enhance employees’ green and creative attitude and behavior.

## Data Availability Statement

The raw data supporting the conclusions of this article will be made available by the authors, without undue reservation.

## Ethics Statement

The studies involving human participants were reviewed and approved by the University of Science and Technology of China Ethics Committee. The patients/participants provided their written informed consent to participate in this study.

## Author Contributions

JW, DC, and WC: conceptualization. JW: methodology. ZB: software and formal analysis. JW, DC, and ZB: validation. WZ: investigation and writing—original draft preparation. JW and DC: resources and project administration. WC: visualization. DC: supervision. All authors have read and agreed to the published version of the manuscript.

## Conflict of Interest

Authors JW, ZB, TS and WZ were employed by the company Anhui Ance Think Tank Consultancy Co., Ltd. The remaining authors declare that the research was conducted in the absence of any commercial or financial relationships that could be construed as a potential conflict of interest.

## Publisher’s Note

All claims expressed in this article are solely those of the authors and do not necessarily represent those of their affiliated organizations, or those of the publisher, the editors and the reviewers. Any product that may be evaluated in this article, or claim that may be made by its manufacturer, is not guaranteed or endorsed by the publisher.
